# Primary intratesticular rhabdomyosarcoma in children: a case report and review of the literature 

**DOI:** 10.1186/s13256-020-02599-z

**Published:** 2021-01-31

**Authors:** James J. Yahaya, Alex Mremi

**Affiliations:** 1 Department of Biomedical Science, College of Health Sciences (CHS), The University of Dodoma, P. O. Box 395, Dodoma, Tanzania; 2 Department of pathology, Kilimanjaro Christian Medical Centre (KCMC), Moshi, Tanzania

**Keywords:** Intratesticular, Rhabdomyosarcoma, Children, Case report

## Abstract

**Background:**

The importance of this paper is to help to emphasize the importance of chemotherapy for children with pure intratesticular rhabdomyosarcoma after radical inguinal orchiectomy is done as first treatment of rhabdomyosarcoma. The information provided in this paper about the follow-up outcomes of the patient described in this paper, it highlights that, recurrence and even metastasis of intratesticular rhabdomyosarcoma in children are more likely to occur if surgery it not combined with chemotherapy.

**Case presentation:**

Herein, we present a 6-year old African male child with a 3 months history of a painless right intratesticular tumour. The tumour was poorly vascularized and was in continuity with the spermatic cord. Pelvic computer tomography (CT) scan showed a heterogeneous mass with well-defined margins without microcalcification and multiple bilateral inguinal enlarged lymph nodes were noticed without pelvic lymphadenopathy. The tumour measured 3.8 × 2.8 × 3.9 cm. The tumour marker panel showed: lactate dehydrogenase of (472 UI/l), alpha-fetoprotein (1.43 UI/ml) and human chorionic gonadotrophin beta (2.9 mUI/ml). Microscopically, the tumour was composed of small to medium size undifferentiated cells. These were oval to spindle, hyperchromatic cells to stromal myxoid degeneration were noted. Tunica albuginea and rete testis both were infiltrated by tumour. The tumour showed high mitotic count which measured 50 mitoses per 10 High Power Field (HPF). The diagnosis of rhabdomyosarcoma (RMS) was confirmed by immunohistochemistry (IHC) testing using myoD antibody which showed strong and diffuse intranuclear staining of the tumour cells. Currently, he is on cyclophosphamide and vincristine chemotherapy regime and his condition has improved much.

**Conclusions:**

The experience obtained from the index case is crucial for the management of patients with intratesticular rhabdomyosarcoma which should always make sure that radical inguinal orchiectomy is covered by chemotherapy and/or radiotherapy. This will potentially lower the possibilities of recurrence and/or metastasis of the tumour, hence improving the prognosis of the patients. We report the clinical, radiological, and laboratory characteristics as well as the outcome of the patient.

## Background

Rhabdomyosarcoma (RMS) is a common mesenchymal malignancy in the pediatric population with a slight male preponderance [1, 2]. In 2013, the World Health Organization (WHO) report on classification of RMS includes four histological subtypes namely alveolar, embryonal, pleomorphic and sclerosing or spindle cell types of RMS [3]. It is estimated that embryonal RMS represents about 70% of all childhood RMS especially those below 10 years of age [4, 5]. Of the head and neck regions, the orbit is the commonest site for embryonal RMS. RMS of the testis, epididymis, and spermatic cord are rare malignant tumours that tend to be encountered sporadically globally [6].

Primary intratesticular rhabdomyosarcoma (PITRMS) is a rare intrascrotal tumour localized in the tunica vaginalis, epididymis, or spermatic cord. Diagnosis of PITRMS can be done on a high degree of clinical suspicion with the help of imaging diagnostic tests, biopsy and immunohistochemistry [4, 7]. PITRMS should be differentiated from sarcomatous germ cell tumours, other intratesticular sarcomas and paratesticular RMS [8, 9]. Immunohistochemical markers are critical to exclude other intratesticular spindle cell sarcomas and germ cell tumors that also present with rhabdomyoblastic differentiation [9]. Little is known about the underlying etiology and stimulus that induce the tumuor growth. Genetic factors, occasional presence of the tumuor at birth and the association of the disease with other neoplasms in the same patient have been reported as predisposing factors [10]. Over the past years, there has been observed a gradual but significant improvement in survival for patients with intratesticular RMS, despite its high grade of malignancy [11]. These results are due to multidisciplinary treatment approaches including surgery, radiotherapy and especially chemotherapy. RMS is a highly chemosensitive neoplasm, and the role of this therapeutic approach has also been clearly demonstrated in the adjuvant setting [5, 10].

Herein, we report the case of a 6-year old African male child who was diagnosed with pure intratesticular RMS of the embryonal variant. This is extremely rare with an incidence of only 1% [4]. We decided to report this case because it will add information on the issue of recurrence when patients are not treated with chemotherapy and/or radiotherapy after radical inguinal orchiectomy as it was in our case.

## Case presentation

A 6-year old African male was admitted in the pediatric ward with a 3-month history of a right testicular mass which was painless. He had no history of trauma, difficulty in passing urine or blood in urine. The parents denied any history of cancer in the family. His past medical, social, family and environmental history were uneventful. Either it was reported that, the patient received Diphtheria, Tetanus, Pertussis and Haemophilus influenza and Hepatitis B, pneumo-conj, and Measle-Rubella (MR) vaccines at the right schedules. On admission, physically, the patient was ill-looking, pale and febrile (39.2 °C). His weight and height were 20.2 kg and 86 cm, respectively with pulse rate and blood pressure of 68 beats per minute and 127/73 mmHg, respectively. The right testis was swollen, painless, and non-tender, nodular and firm in consistency. The overlying scrotal skin was shining with engorged overlying blood vessels. His neurological examination revealed normal motor function (normal gait and muscle tone), normal sensory function and normal reflex responses.

**Fig. 1:**
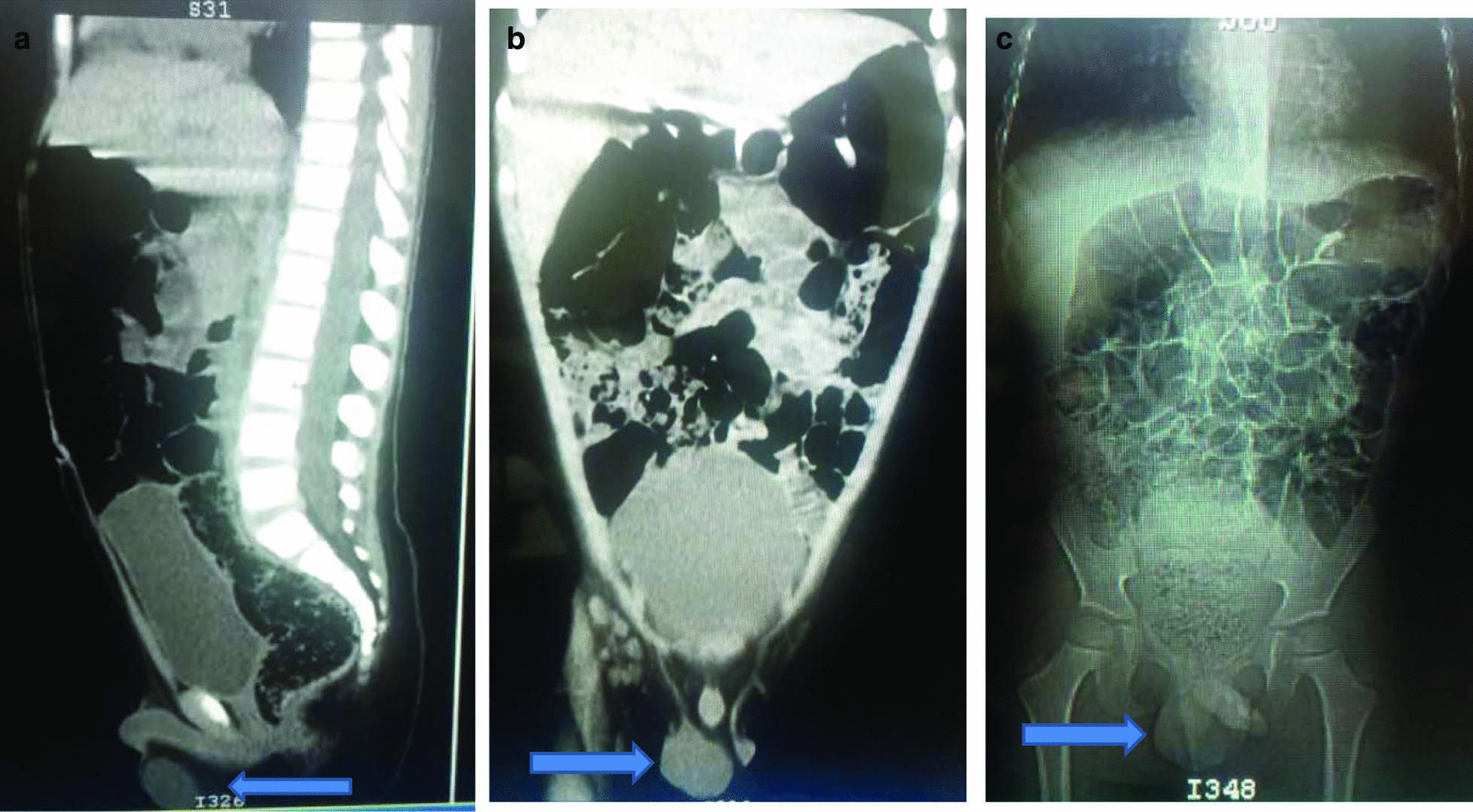
Computer tomography scan images for sagittal (**a**), coronal (**b**) and Scout views (**c**). The arrow is pointing to a well defined homogenous mass replacing the right testis for images **a** and **b** while the left testis and the penile shaft were preserved particularly for image **c**

The laboratory tests included the following: haemoglobin level 8.3 gm/dl (low), aspartate transaminase (AST) 10-36 IU/l (normal), alanine transaminase (ALT) 36-39.1 IU/l (normal), serum creatinine 0.81 mg/dl (normal), protein in urine 3.1 gm/ml (normal), human immunodeficiency virus (HIV) and hepatitis B surface antigen (HBsAg) were both negative. Pelvic computed tomography (CT) scan showed a heterogeneous mass with well-defined margins without microcalcification (Fig. 1). Multiple bilateral inguinal enlarged lymph nodes were noticed. There was no ascites or intraperitoneal free air. Besides, there was no pelvic lymphadenopathy.

The tumour was poorly vascularized and was in continuity with the spermatic cord. The tumour measured 3.8 × 2.8 × 3.9 cm. The left testis was normal in size and orientation. The panel of tumour markers included: lactate dehydrogenase of (472 UI/l), alpha-fetoprotein (1.43 UI/ml) and human chorionic gonadotrophin beta (2.9 mUI/ml). The patient’s tumour was staged as clinical stage I. By using the criteria for risk stratification of patients with RMS established by the Children’s Oncology Group for Soft-Tissue Sarcoma (COG-STS) committee [12] which adopted data from the Intergroup Rhabdomyosarcoma Study (IRS) -III and IRS-IV; the patient was regarded to be in low risk group (group I) by having clinical stage 1 and embryonal histopathological type.

The pelvic, abdominal and chest CT scans showed no metastases to either lungs or mediastinum. The patient had operation (orchidectomy). During the operation, external oblique aponeurosis was opened; ilio-inguinal nerve was isolated and secured. Inguinal canal contents were dissected. There was no vas deferens that was seen and the left testis was infiltrated by the tumour and the tumour had gone beyond the tunica vaginalis. A straw colored jelly-like material was seen surrounding the tumour within the scrotal sac.

**Fig. 2:**
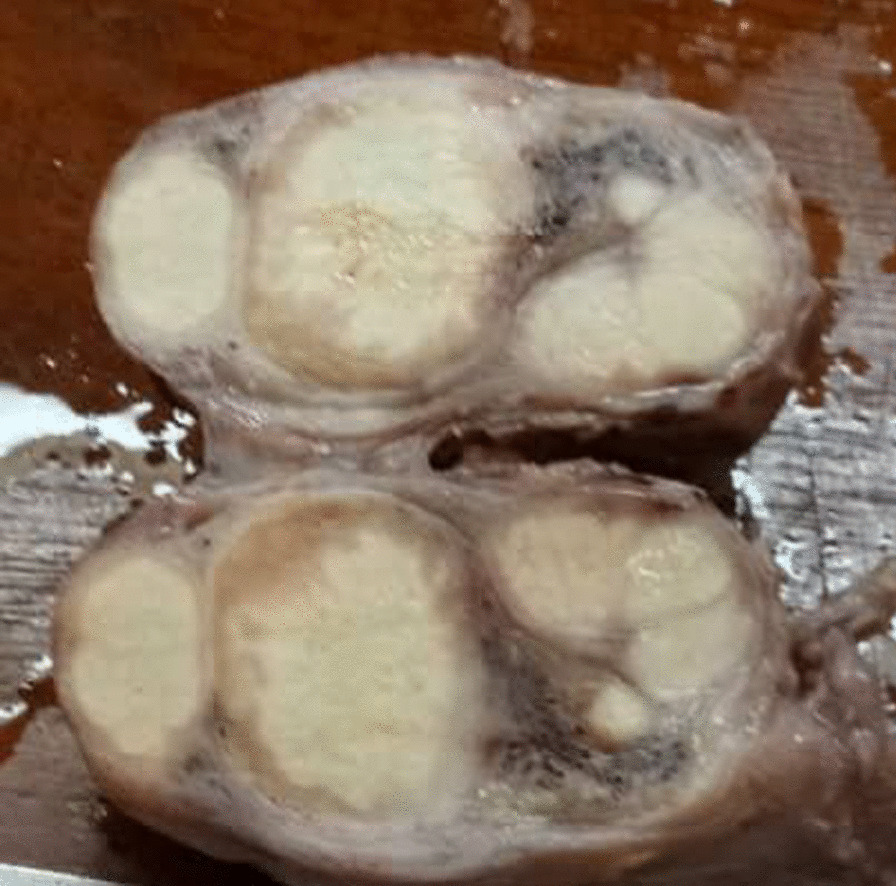
Gross appearance of the testicular mass. The tumour is white-tan, lobulated and is growing by pushing into the tunica vaginalis

The dissected inguinal canal contents were clamped, ligated with vicryl 3/0 and transected. The malformed testis by the infiltrating mass was dissected and excised and hemostasis was achieved. The specimen was taken for histological evaluation. Retroperitoneal lymph node dissection was not performed in our case as radiologically there was no lymphadenopathy. Macroscopically, the submitted specimen comprised of a grayish-yellow fibro-fatty tissue that measured 4 × 3 × 2 cm. The cut surface showed a lobulated white-tan and firm mass (Fig. 2). There was no necrosis or haemorrhage.

**Fig. 3:**
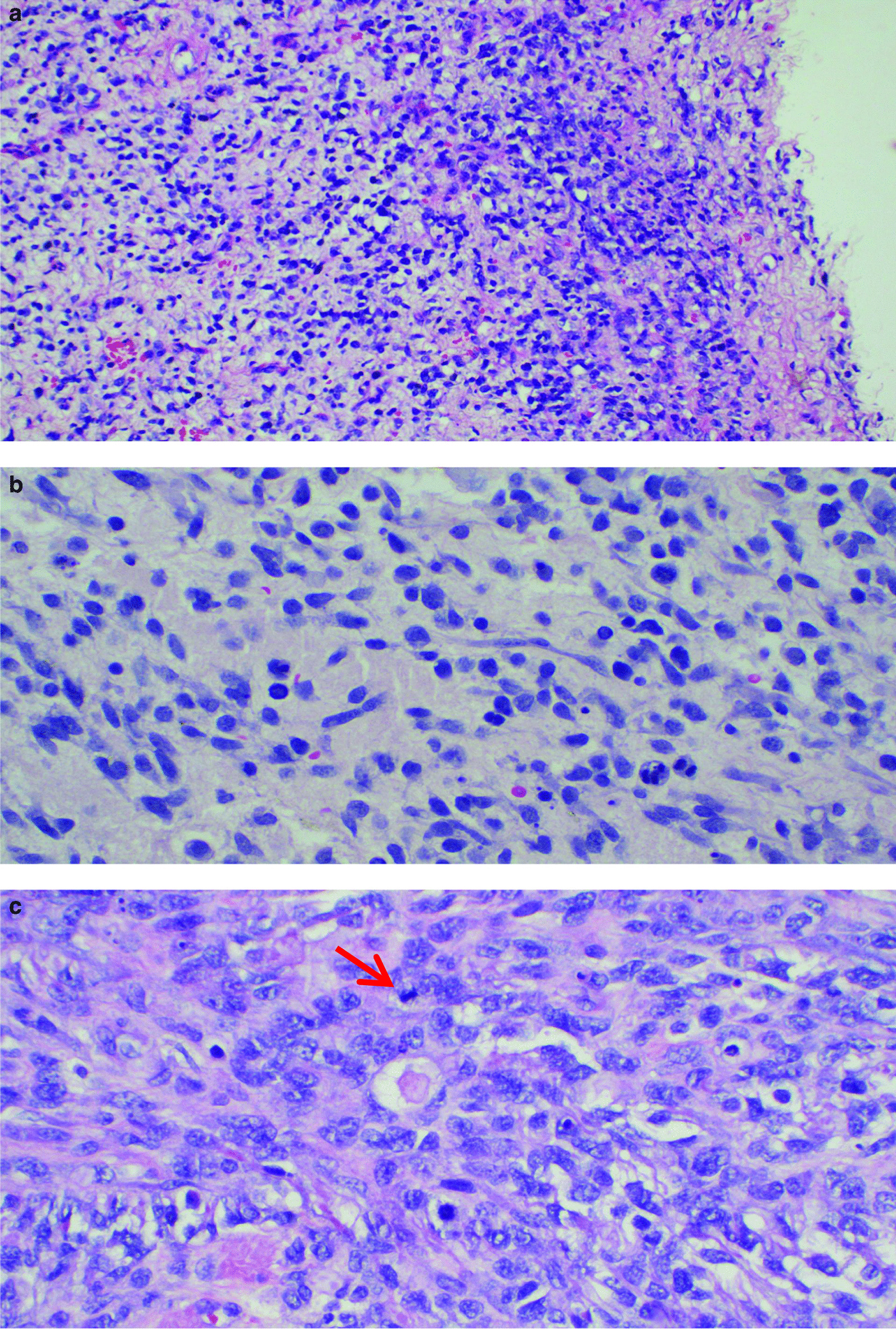
**a** Infiltration of the testicular tissue by the tumour cells and proliferation of vascular structures. The tumour growth is diffuse and the cells are spindle (haematoxylin and eosin stain, ×400). **b** The tumour cells are spindle and roundish with hyperchromatic (haematoxylin and eosin stain, ×200). **c** Proliferation of rhabdomyoblast cells. The cells are hyperchromatic, pleomorphic with irregular coarse nuclear chromatin and abundant eosinophilic cytoplasm. The arrow is pointing to numerous brisk mitotic figures were evident (haematoxylin and eosin stain, ×400)

**Fig. 4:**
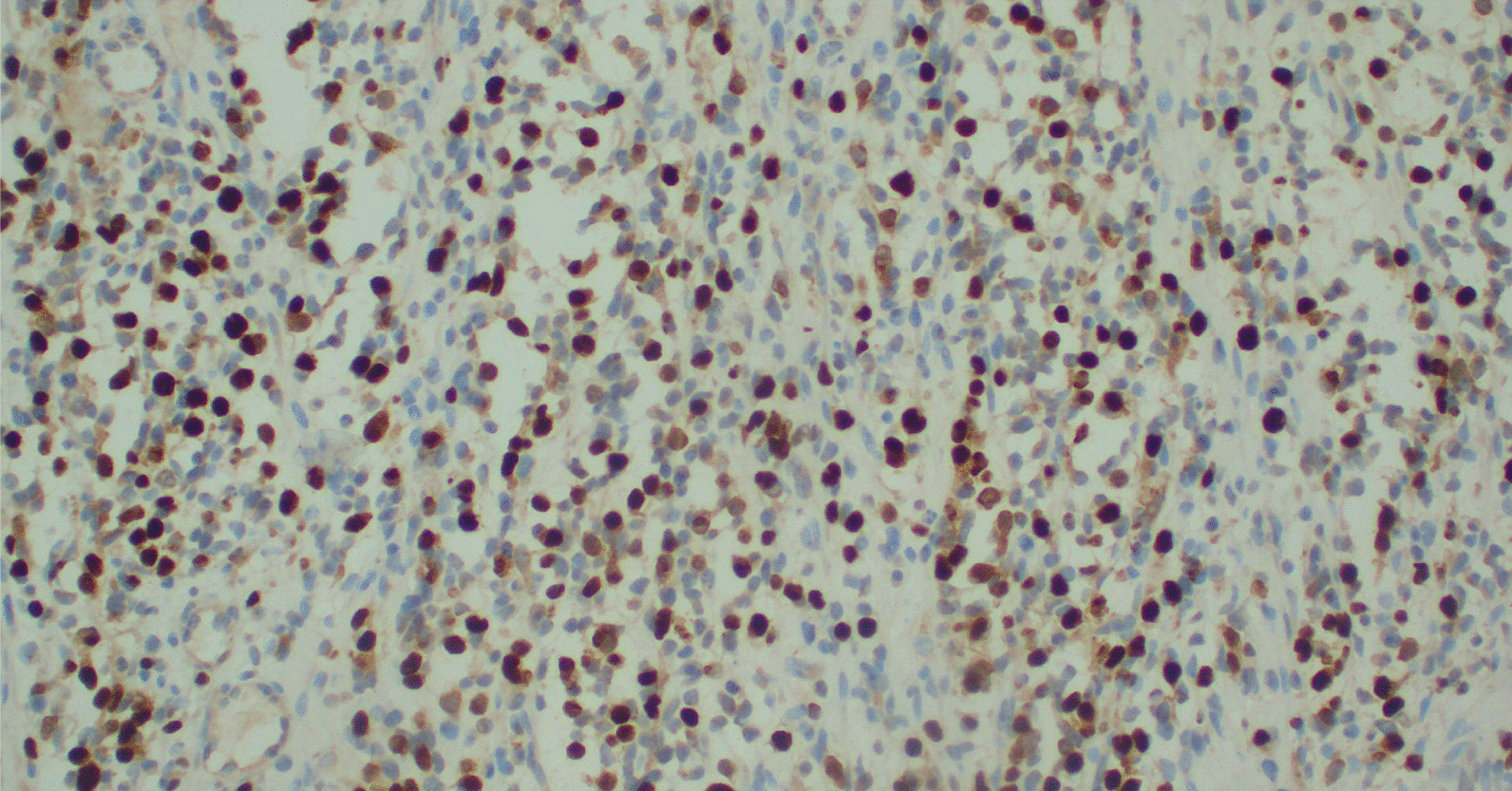
immunohistochemistry (IHC) staining of the tumour cells by myoD1 antibody. The tumour cell are diffusely and strongly staining showing intranuclear staining (IHC staining, ×200)

Microscopically, the tumour was composed of the presence of small to medium size undifferentiated cells (Fig. 3a–c). These were oval to spindle, hyperchromatic cells with focal stromal myxoid degeneration were noted. Tunica albuginea and rete testis both were infiltrated by tumour. The tumour showed a high mitotic count which measured 50 mitoses per 10 high power field (HPF). The diagnosis of embryonal RMS was confirmed using myoD1 antibody which showed strong and diffuse intranuclear staining of the tumour cells (Fig. 4).

The tumour recurred five months after radical inguinal orchiectomy (RIO) had been done because the patient delayed to start chemotherapy on time until when the parents saw a mass which was gradually increasing in size. Then he was taken back to hospital where he was given chemotherapy after evaluation with chest CT scan to rule out for metastasis which revealed normal findings. The chemotherapy agents used were 1.2 mg iv cyclophosphamide, 0.94 mg iv actinomycin D and 1032 mg iv vincristine which he completed after 5 months. We followed-up the patient for one year and his postchemotherapy period was uneventful. Currently, the child is healthy and he is continuing with schooling.

## Discussion

This paper presents the case of a 6-year old African male with primary intratesticular RMS of embryonal variant which was localized. However, it recurred following radical inguinal orchiectomy after delaying to start chemotherapy. This case is unique in the sense that the patient is relatively younger compared to majority of the cases reported in the English literature. The other unique feature of this patient is that the patient falls in the low risk group based on the risk stratification of RMS.

The histogenesis of PITRMS is still enigmatic. It has been theorized that, either PITRMS originates from dedifferentiated mesenchyme having the capacity for rhabdomyoblastic differentiation, or perhaps from embryonal muscle tissue that has been displaced during the early stages of tissue development, but not from metaplasia of the connective tissue or smooth muscle [13]. Another school of thought is that, the sarcomatous component seen in germ cell tumours, may be related to the development of PITRMS [14]. PITRMS constitutes the group of intrascrotal tumours and other types of intrascrotal masses are paratesticular RMS.

Intrascrotal RMS can either be intratesticular or paratesticular. PITRMS are very rare and they comprise less than 1% of all the testicular sarcomas whose prevalence is 2% of all testicular tumours. On the other hand, studies have reported that, paratesticular RMS have an incidence of approximately 7% of the intrascrotal RMS [7, 15]. Primary testicular sarcoma is an infrequently reported and rare indolent tumour with the potential for distant metastases [16]. A diagnosis should only be made after exclusion of the more commonly seen paratesticular smooth muscle tumours [17].

PITRMS clinically presents as painless intrascrotal masses which may progress for a few weeks and become painful and a few cases have been reported to present with pain. Similarly, trauma is associated in only 7% of cases and is not thought to be causative but rather to draw attention to the tumour [13]. Usually, PITRMS patients have a slow growing intrascrotal mass for the first six months before diagnosis. The mean age of patients with PITRMS is approximately 30 years [15, 18]. The tumour size may increase up to 7 cm in diameter [17].

Diagnosis of PITRMS is based on different approaches. This includes imaging (ultrasound, CT scan and MRI), IHC staining, gross appearance and histopathology. Scrotal ultrasound and abdominal CT scan may be used to determine an intratesticular or paratesticular origin of the scrotal mass [16]. Ultrasound is the most common used imaging technique for examining testicular mass and adjacent organ. IHC markers are critical for excluding other intratesticular spindle cell sarcomas and germ cell tumours that also present with rhabdomyoblastic differentiation. RMS exhibit positivity for SMA, S-100 and vimentin and negative for cytokeratin [18]. Tumour markers in PITRMS including AFP, HCG and LDH are typically within normal ranges [16].

Treatment of PITRMS still is not comprehensive and trials have not been explorative due to limited availability of cases because of the rarity nature of the disease. A multidisciplinary therapeutic approach which involves surgery and chemotherapy with or without radiotherapy has been established based on risk stratified groups [7]. Radical inguinal orchiectomy (RIO) has been found the mainstay choice of treatment which is approached by making a wide incision and high ligation of the spermatic cord and the testes in order to minimize the possibility of a residual tumour in the course of treatment [19].

From the observations in the IRS-III, three recommendations were put forward which include (1) Treatment for children with RMS should be based entirely on risk stratification, site of the primary tumour site and the extent of disease (2) Children with low risk groups (group I or II) tend to respond well to RIO and chemotherapy (3) Radiotherapy should be provided for children with risk group II only [7]. This treatment regimen is also effective for patients with a favourable histological type of the head and all other patients seem to require more intensive multimodality therapy. Surgical resection with chemotherapy was reported to be an effective way to achieve better clinical outcomes and long-term survival in adult patients with metastases from PITRMS [20].

Retroperitoneal node clearance is controversial and is probably not justified for staging or in initial treatment, but it has a role in debulking disease if nodes persist after chemotherapy [13]. There is a greater probability of retroperitoneal disease in adults with PITRMS, and as a result retroperitoneal lymphadenectomy is recommended in these cases. It is necessary in the case of adults, even those with disease-free lymph nodes showing up in pre-operative imaging studies [21]. It has been advocated that, where the tumour is unresectable, incisional biopsy for confirming the diagnosis followed by chemotherapy and/or radiotherapy [22].

Prognosis of PITRMS is said to be poor due to its aggressiveness nature of the tumour. A number of prognostic factors determine the clinical outcomes of any patient with rhabdomyosarcoma including those with PITRMS. Age of the patient at diagnosis, histological type, retroperitoneal lymph node involvement (RPLI), risk group, site of the primary tumour, intensification of treatment and metastasis altogether play a major role in prognostication of the disease for children, adolescents and adults [7, 11, 22]. In the intergroup rhabdomyosarcoma study-III (IRS-III) which was conducted from 1984 to 1991 reported that histological type, clinical group and intensification of treatment were the predictors of both progression free survival and 5-year overall survival rate of the children with RMS [7]. However, in the same study it was found that, for patients with metastasis (clinical group IV), intensification including alteration of chemotherapy drugs [2, 4]. Sites of the primary tumour such as orbit, head, pelvic area, intratesticular, meningeal and paratesticular have also been reported to carry poor prognosis when compared to the rest of the other body parts [2].

The use of molecular tests in complementing the process of determining the prognosis of patients with RMS seems to provide promising results. For example, Pappo *et al.* reported that tumour-cell hyperdiploidy correlates with a favorable outcome in children and young adults with embryonal RMS [4]. Also Douglas *et al.* reported that *t*(2; 13) characterizes alveolar RMS with a poorer prognosis [23]. Molecular cloning of the *t*(2;13) breakpoints has been reported to identify a fusion gene, *PAX3-ALV*, that can now be readily identified by the reverse polymerase chain reaction [24]. Lack of potential and novel targeted therapies for RMS that are validated for treatment of RMS, is still a challenge in improving the prognosis of patients with RMS. Promising results from different studies have reported results in experimental animals which are promising and will be of benefits to the patients in the near future.

## Conclusions

From the observation of the case presented in this paper, it may be learned that, radical inguinal orchiectomy should always be followed immediately with adjuvant chemotherapy in order to prevent recurrence of the resected primary tumour as it was the experience in our patient. Also the use of ancillary tests such as molecular prognostic factors would be of paramount importance in improving the clinical outcomes of children as well as adults diagnosed with PITRMS.

## Data Availability

All data and materials are available for sharing if needed.

## Abbreviations

PITRMS: Primary intratesticular rhabdomyosarcoma
RMS: Rhabdomyosarcoma
IRS-III: Intergroup rhabdomyosarcoma study-III
IRS-IV: Intergroup rhabdomyosarcoma study-IV
RPLI: Retroperitoneal lymph node involvement
RIO: Radical inguinal orchiectomy
HPF: High power field
IHC: Immunohistochemistry
WHO: World Health Organization
CT: Computed tomography
